# Dairy Intake and Acne Vulgaris: A Systematic Review and Meta-Analysis of 78,529 Children, Adolescents, and Young Adults

**DOI:** 10.3390/nu10081049

**Published:** 2018-08-09

**Authors:** Christian R. Juhl, Helle K. M. Bergholdt, Iben M. Miller, Gregor B. E. Jemec, Jørgen K. Kanters, Christina Ellervik

**Affiliations:** 1 Department of Biomedical Sciences, Faculty of Health and Medical Sciences, University of Copenhagen, 2100 Copenhagen, Denmark; christian.r.juhl@gmail.com; 2 Department of Production, Research, and Innovation, Region Zealand, 4180 Sorø, Denmark; hellebergholdt@hotmail.com; 3 Department of Dermatology, Zealand University Hospital, 4000 Roskilde, Denmark; miller@dadlnet.dk (I.M.M.); gbj@regionsjaelland.dk (G.B.E.J.); 4 Department of Clinical Medicine, Faculty of Health and Medical Sciences, University of Copenhagen, 2100 Copenhagen, Denmark; 5 Department of Laboratory Medicine, Boston Children’s Hospital, 300 Longwood Avenue, Boston, MA 02115, USA; 6 Department of Pathology, Harvard Medical School, Boston, MA 02115, USA

**Keywords:** meta-analysis, dairy, milk, acne, yogurt

## Abstract

A meta-analysis can help inform the debate about the epidemiological evidence on dairy intake and development of acne. A systematic literature search of PubMed from inception to 11 December 2017 was performed to estimate the association of dairy intake and acne in children, adolescents, and young adults in observational studies. We estimated the pooled random effects odds ratio (OR) (95% CI), heterogeneity (*I*^2^-statistics, *Q*-statistics), and publication bias. We included 14 studies (*n* = 78,529; 23,046 acne-cases/55,483 controls) aged 7–30 years. ORs for acne were 1.25 (95% CI: 1.15–1.36; *p =* 6.13 × 10^−8^) for any dairy, 1.22 (1.08–1.38; *p =* 1.62 × 10^−3^) for full-fat dairy, 1.28 (1.13–1.44; *p =* 8.23 × 10^−5^) for any milk, 1.22 (1.06–1.41; *p =* 6.66 × 10^−3^) for whole milk, 1.32 (1.16–1.52; *p =* 4.33 × 10^−5^) for low-fat/skim milk, 1.22 (1.00–1.50; *p =* 5.21 × 10^−2^) for cheese, and 1.36 (1.05–1.77; *p =* 2.21 × 10^−2^) for yogurt compared to no intake. ORs per frequency of any milk intake were 1.24 (0.95–1.62) by 2–6 glasses per week, 1.41 (1.05–1.90) by 1 glass per day, and 1.43 (1.09–1.88) by ≥2 glasses per day compared to intake less than weekly. Adjusted results were attenuated and compared unadjusted. There was publication bias (*p =* 4.71 × 10^−3^), and heterogeneity in the meta-analyses were explained by dairy and study characteristics. In conclusion, any dairy, such as milk, yogurt, and cheese, was associated with an increased OR for acne in individuals aged 7–30 years. However, results should be interpreted with caution due to heterogeneity and bias across studies.

## 1. Introduction

Acne is a common chronic inflammatory skin disease of sebaceous follicles [1,2]. Clinically, acne is characterized by the presence of open and closed comedones, papules, pustules, and dermal tissue damage with eventually heavy scar formation. Follicular hyperkeratosis, modifications of the sebofollicular microbiome, increase production of sebum with increased amounts of pro-inflammatory monounsaturated fatty acids, and Th17-cell-mediated inflammatory responses are all involved in acne pathogenesis. Sebum production can be induced by insulin-like growth factor-1 (IGF-1) and androgens, whose adrenal and gonadal synthesis is stimulated by IGF-1 [3]. Although prevalence varies across studies, acne is common in children and adolescents aged 12–24 years and is moderate to severe in 15–20% of cases [1,4,5,6].

Heritability of acne alone does not explain high acne prevalence rates of over 80% in western countries [5,7]. It has long been debated if a Western diet *per se* or specific dietary components contribute to the prevalence and severity of acne [4,8]. This has predominantly been investigated in observational studies and only a few trials exist [9]. In particular, dairy products have been incriminated. Milk-derived amino acids promote insulin secretion and induce hepatic insulin-like growth factor-1 (IGF-1) synthesis [10]. IGF-1 has been suggested as the pivotal driver of acne and stimulates follicular epithelial growth and keratinization [11,12,13]. IGF-1 gene polymorphism has been shown to increase susceptibility to acne [14] and IGF-1 plasma levels correlate with acne severity [12]. 

Several worldwide observational studies have been published on dairy intake and acne in children, adolescents, and young adults (7–30 years) in various countries [15,16,17,18,19,20,21,22,23,24,25,26,27]. Some narrative and systematic reviews about dairy intake and acne have been published [4,9,28]. Recently, a meta-analysis of dairy and acne was published [29] but with several methodological flaws, including lack of bias assessment and inadvertent double-counting of studies due to duplicate publications [19,23,30,31] that caused inappropriate weighting of results and skewed pooled estimates. So far, no previous meta-analysis has statistically combined the observational studies in an attempt to estimate the effect of the association of dairy intake and acne with the heterogeneity across studies, a bias assessment, a stratified analysis by study characteristics, and publication bias. 

The primary objective of this study was therefore to perform a meta-analysis to estimate the association of acne in children, adolescents, and young adults consuming any dairy products. Furthermore, our aim was to explore the association between acne and intake of varies types of dairy (milk, yogurt, cheese), dairy subgroups (full fat, low fat, skim), and various amounts and frequencies of dairy intake (times per week or day). 

## 2. Methods

This systematic review and meta-analysis was undertaken according to Meta-analysis of Observational Studies in Epidemiology (MOOSE) guidelines and according to a specified protocol (Supplementary Materials). The search, selection of studies, full-text reading, and data extraction were performed by CRJ and verified by CE.

### 2.1. Search Strategy

The search was performed on 11 December 2017 and included all studies up until that date. Studies were identified in the PubMed database using the search terms: (“Dairy products”[Mesh] OR dairy[All Fields] OR milk[Mesh] OR milk[All Fields] OR yogurt[All Fields] OR cheese[All Fields] OR lifestyle[All Fields]) AND (“Acne Vulgaris”[Mesh] OR Acne[All Fields]). We identified 241 records. 

### 2.2. Eligibility Criteria

All observational studies (case-control, cross-sectional, population-based, retrospective) on childhood, adolescent, or young adult acne (max age of 30 years) were eligible if they reported a risk estimate and a 95% confidence interval for acne in a dairy group vs. a non-dairy group, or the raw numbers from 2 by 2 tables of dairy intake and acne. 

### 2.3. Procedure for Selection of Studies

**Figure 1:**
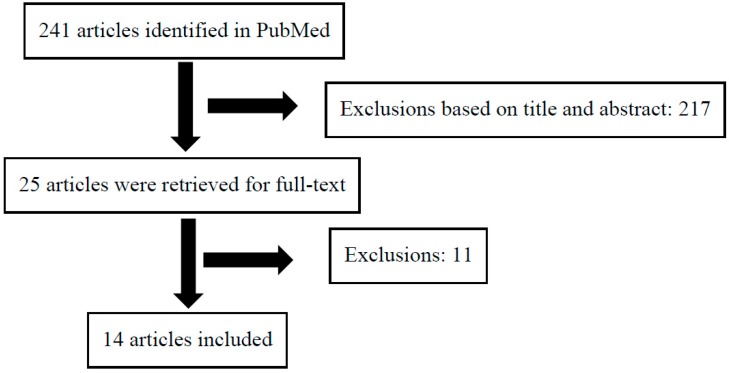
Flow diagram for meta-analysis.

We screened the title and abstracts of 241 articles (Figure 1). If relevant, we retrieved the full-text articles. We identified 25 full-text articles, but excluded the following 11 studies: duplicate [19] (there was a statement in the article by Grossi that it was the same cohort and results as [23]), beliefs/opinions about acne aggravating food items [32,33], semi-fat/whole milk vs. skim milk/no milk drinkers [34], Chinese ying-yang medicine [35], no control group [36], adult acne (mean age ≥ 30 years) [37,38], milk as part of a Mediterranean diet [39], milk only as a continuous variable in acne and non-acne groups [40], and poorly defined intake [41]. In total, we included 14 studies. Two other studies were identified outside the search, but these studies were duplicates and published simultaneously without a clear statement of which one was the original; therefore, we did not include these papers [30,31]. The study selection process is shown in a flow diagram (Figure 1).

### 2.4. Data Extraction and Management

We extracted the following data for each study and entered the information in an excel spreadsheet: author, year, population, country, age, gender, study design, how outcome was estimated, dairy type (dairy, milk, yogurt, cheese), dairy subtype (whole (full-fat), low-fat, skim), dairy amount, frequency of intake (times per day or week), numbers of acne patients and controls subjects in each category of dairy intake, crude and/or adjusted odds ratio (OR) or prevalence ratio with 95% confidence interval (CI), raw numbers to calculate crude OR (95% CI). 

### 2.5. Overall and Subgroup Analyses

The primary objective was to perform a meta-analysis to estimate the odds ratio of acne in children, adolescents, and young adults consuming any dairy compared to those who do not. The secondary objective was to estimate the odds ratio of acne associated with intake of varies types of dairy (milk, yogurt, cheese), dairy subgroups (full-fat, low-fat, skim), and various amounts and frequencies of dairy intake (times per week or day) compared to those who did not consume any dairy/milk.

### 2.6. Risk of Bias and Study Quality Assessment

The quality of each study was evaluated and scored using the nine-star Newcastle-Ottawa Scale (NOS), a tool used for quality assessment of nonrandomized studies [42]. Studies were evaluated based on selection, comparability, exposure, and outcome, and scored by a maximum of nine points. Scores above five indicate moderate to high study quality. The NOS for cohort and case-control studies was retrieved from [43].

### 2.7. Statistical Analyses

The meta-analyses were performed with STATA SE 14.0 (Stata Corp., College Station, TX, USA). Using raw numbers, we calculated the crude odds ratios OR (95% CI). Analyses were performed for any dairy intake, any milk intake, full-fat dairy, whole milk, and low-fat/skim milk compared to those who did not consume any dairy/milk (study specific definitions). For any milk intake, whole milk and low-fat/skim milk, analyses of frequencies (times per week or day) were performed using studies by Adebamowo et al. [15,16,17], as these studies had identical ascertainment of the frequency of milk intake. DerSimonian and Laird (D + L) pooled random effects estimates were used. We also present inverse variance (I-V) fixed effects in supplementary Figures. Heterogeneity was assessed by Cochrane Q statistic test and *I*^2^-statistical analysis. The *I*^2^-statistical analysis assess what proportion of the observed variance reflects variance in true effect sizes rather than sampling error [44]. Publication bias was examined visually by funnel plots and statistically using Egger’s test (one-sided) [45] and by using the Duval and Tweedie's Trim and Fill to simulate where potential unpublished studies would belong in the funnel plot and to calculate a hypothetical new pooled odds ratio based on the added simulated studies. Robustness of the meta-analysis was examined by “leaving-one-out” analysis. Publication bias and robustness were carried out by use of the statistical program Comprehensive Meta-Analysis (CMA) version 3 (Biostat, Englewood, NJ, USA) for any dairy intake vs. no dairy intake and any milk intake vs. no milk intake. Four studies provided adjusted estimates for milk intake, with one study providing them as odds ratios [46], and three studies as prevalence ratios [15,16,17]. In a sensitivity analysis, we used only adjusted prevalence ratios from the studies by Adebamowo et al. [15,16,17]. Stratification on acne severity was not possible because of too few studies. 

## 3. Results

### 3.1. Description of the Studies

In total, 14 studies were eligible. Figure 1 shows the flow diagram of the selection of articles for the meta-analysis. The studies were published in 2005–2017 and included a total of 78,529 individuals of which 23,046 had acne and 55,483 were controls (Table 1). The prevalence of acne ranged from 7–89% in population studies and 36–83% in case-control studies. Two studies used non-acne dermatological controls [23,26] and the rest used healthy controls. Five studies were cross-sectional [20,21,27,46,47], five studies were case-control [18,22,23,24,26], one study was retrospective [15], and three studies were longitudinal [16,17,25]. The age-group ranged from 7–30 years. Two studies were only in females [15,17], three studies only in males [16,26,46], and the rest included both males and females. The studies covered five continents: Africa [21], Asia [18,24,47], Europe [20,22,23,25,26,27], North America [15,16,17], and South America [46]. Four studies included less than 1000 individuals in total [18,21,22,23,47], whereas the rest ranged from 1285 to 46,879 individuals (Table 1). Four studies used the Willet food frequency questionnaire [15,16,17,21]. In six studies, acne was self-reported in a questionnaire [15,16,17,20,25,27], and in eight studies, acne was a physician verified diagnosis [18,21,22,23,24,26,46,47]. Five studies provided adjusted estimates, including four on milk intake and one on dairy, two of the studies reported odds ratios, and three studies reported prevalence ratios [15,16,17,25,46]. The reference group varied among the articles and included not weekly [15,16,17,18,25], not daily [20,21,46], never [23,27], and unclear [24,26,47].

### 3.2. Findings

**Figure 2:**
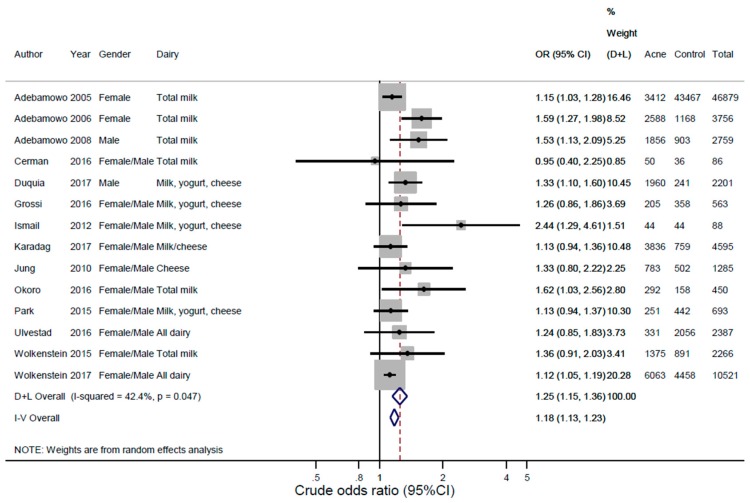
Meta-analysis of dairy intake and acne vulgaris: individual studies. The figure shows the individual studies and the unadjusted pooled random effect estimate from the meta-analysis of dairy intake and acne vulgaris. *I*^2^(%): *I*-square heterogeneity expressed as percentage. *p*-value(het): *p*-value from Cochran’s *Q*-statistic assessing heterogeneity. D + L: DerSimonian and Laird pooled random effects estimates. See Table 1 for references.

**Figure 3:**
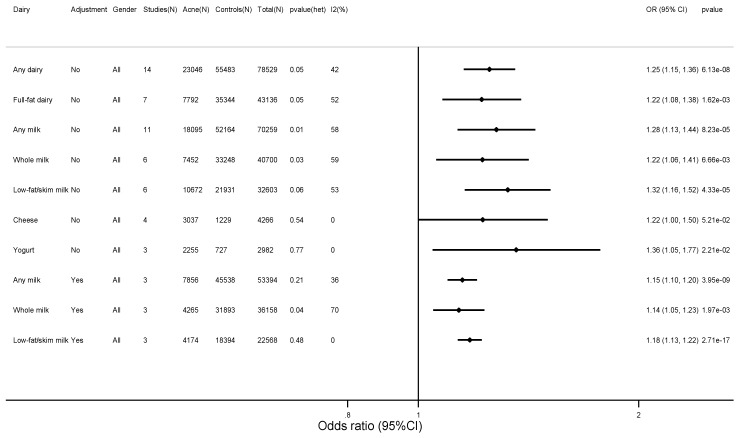
Meta-analyses of dairy intake and acne vulgaris: summary estimates. The figure shows the unadjusted pooled random effects estimates from each of the meta-analyses, which can be found in the supplementary material. *I*^2^(%): *I*-square heterogeneity expressed as percentage. *p*-value(het): *p*-value from Cochran’s *Q*-statistic assessing heterogeneity.

Random effects pooled unadjusted odds ratios for acne were 1.25 (95% CI: 1.15–1.36; *p =* 6.13 × 10^−8^) for any dairy (Figure 2), 1.22 (1.08–1.38; *p =* 1.62 × 10^−3^) for full-fat dairy, 1.28 (1.13–1.44; *p =* 8.23 × 10^−5^) for any milk, 1.22 (1.06–1.41; *p =* 6.66 × 10^−3^) for whole milk, 1.32 (1.16–1.52; *p =* 4.33 × 10^−5^) for low-fat/skim milk, 1.22 (1.00–1.50; *p =* 5.21 × 10^−2^) for cheese, and 1.36 (1.05–1.77; *p =* 2.21 × 10^−2^) for yogurt compared to those who did not consume these food items (Figure 3 and Supplementary Figures S1–S6). 

Random effects meta-analyses for acne by frequency of any milk intake compared to an intake of ≤1 glass of milk per week showed an odds ratio of 1.24 (0.95–1.62) by 2–6 glasses per week, 1.41 (1.05–1.90) by 1 glass per day, and 1.43 (1.09–1.88) by ≥2 glasses per day for any milk; results for whole milk and low-fat/skim milk were close (Supplementary Figures S7–S10).

### 3.3. Sensitivity Analyses, Heterogeneity, Publication Bias, and Qualitative Bias Assessment

The I^2^ heterogeneity ranged from 0–70% (Figure 2). To explore heterogeneity, we stratified the analysis for any dairy intake and acne by age, gender, number of cases, continent, design, acne diagnosis, and reference group (Supplementary Table S1, Supplementary Figures S11–S17). Stratifying by age did not show any differences. Stratifying by gender showed similar odds ratios in males and females, but meta-analyses of females had higher heterogeneity. Stratifying by the number of acne cases showed that larger studies had smaller odds ratios with more narrow confidence intervals, but higher heterogeneity compared to those of the smaller studies, but the confidence intervals were overlapping. Stratifying analyses by continent showed that studies from Europe had the smallest odds ratios, followed by North and South American studies, and with Asian and African studies with the largest odds ratios. Stratifying by design removed heterogeneity and showed that prospective studies had the largest odds ratios. Stratifying by ascertainment of acne diagnosis showed that studies using self-reported acne as an outcome had higher heterogeneity compared to studies with physician verified diagnoses of acne. Stratifying by reference group showed overall similar summary estimates, but with the highest heterogeneity in studies with “less than weekly” being the reference group. The Newcastle-Ottawa qualitative assessment scale of bias with similar items as in the statistical heterogeneity assessments revealed scores of 2–5 in case-control studies [18,22,23,24,26] and 2–6 in cohort studies [15,16,17,20,21,25,27,46,47] out of a potential max of 9 points (Supplementary Table S2). 

Random effects pooled adjusted estimates for any milk, whole milk, and low-fat/skim milk were similar but attenuated compared to their unadjusted estimates (Supplementary Figures S18–S20). 

Leave-one-out analyses for any dairy or any milk intake did not show any gross deviations, but the retrospective study by Adebamowo [15] influenced the summary estimates the most (Supplementary Figures S21–S22). Funnel plot and *p*-value for Egger’s test revealed publication bias for any dairy (*p*-Egger = 4.71 × 10^−3^) (Supplementary Figure S23); Duval and Tweedie’s Trim and Fill method estimated that five studies were missing for “any dairy”, and the imputed point estimate would be 1.16 (1.06–1.28) had these five studies been added. Funnel plot and *p*-value for Egger’s test revealed publication bias for any milk (*p*-Egger = 2.73 × 10^−2^) (Supplementary Figure S24); Duval and Tweedie's Trim and Fill method estimated that one study was missing for “any milk”, and the imputed point estimate would be 1.26 (1.11–1.44) had this study been added.

The New-Castle Ottawa qualitative assessment scale of bias revealed a scores of 2–5 in case-control studies [18,22,23,24,26] and 2–6 in cohort studies [15,16,17,20,21,25,27,46,47].

## 4. Discussion

Intake of any dairy, any milk, full-fat dairy, whole milk, low-fat/skim milk, and yogurt regardless of amount or frequency were associated with a higher odds ratio for acne compared to no intake in individuals aged 7–30 years. Intake of cheese was associated with a borderline higher odds ratio for acne compared to no intake. Stratifying the association of any milk by frequency of intake revealed that intake of 1 glass of milk or more per day was associated with a higher odds ratio for acne, whereas 2–6 glasses per week was not, compared to intake less than weekly. Stratified analyses for any dairy intake and acne fat content demonstrated that full-fat dairy and whole milk had lower odds ratios, whereas low-fat/skim milk had higher odds ratios than the overall summary estimates; a likely explanation for this observation could be that the amount of milk consumed for low-fat/skim milk is higher than that for whole milk. However, results should be interpreted with caution due to heterogeneity and bias across studies.

The meta-analyses showed considerable heterogeneity reflecting the heterogeneous age and gender of the participants, various study characteristics, ascertainment of information about milk intake and acne, reporting of milk intake, and acne severity across the studies. In general, stratifying on subgroups in sensitivity analyses revealed that heterogeneity diminished for most subgroups, but also revealed that especially meta-analyses conducted on females, whole milk, North America, and questionnaire ascertained acne diagnosis demonstrated high heterogeneity. Prospective studies and studies with physician-verified diagnosis of acne had low heterogeneity.

Despite the stratifications, confidence intervals were overlapping. 

Stratifying on age and gender demonstrated similar odds ratios; however, the gender stratified analyses had higher odds ratios than in the gender combined analyses. Smaller studies had higher odds ratios than large studies, African and Asian studies had higher odds ratios than other studies, and prospective designs had higher odds ratios than other designs. A recent multinational European online questionnaire study in adolescents showed that acne prevalence did not differ by gender but differed by country, and acne was more prevalent in younger people and obese people [27]. Intake of milk varies globally and is largely dependent on genetically determined lactase persistency, which is high in people of Northern European descent, but lower in people of Southern European descent, patchy in Africa, and low in the Middle East and Asia [48]. The weaning of the lactase enzyme activity usually happens in childhood and early adolescent years. How the age of weaning of the lactase enzyme activity impacts acne development is not known.

We used random effects method in all meta-analyses, which includes between-study variance and has a higher degree of statistical uncertainty built into the model. Thus, 95% confidence intervals are wider compared to fixed effects models. Even in these models, the results of the meta-analyses were significant. There was evidence of publication bias with Egger’s test with an overweight of smaller studies overestimating the odds ratio compared to the pooled summary estimate. If the meta-analyses had captured all the relevant studies, we would expect the funnel plots to be symmetric. The selective reporting may be explained by studies with null-findings or negative results being deliberately not published because of authors not submitting or editors rejecting them or authors not finding enough merit in a potential publishable study [49]. Furthermore, some studies reported only the pooled exposures for different dairy groups rather than showing the stratified results for each of the dairy groups and/or for each reported frequency of intake [23,25,26], and some studies had only collected an overall dairy or milk variable with no possibility for stratification [20,22]. However, the trim and fill method did not change the overall estimates for “any dairy” or “any milk” remarkably. 

There are many limitations of the included studies [4]. Self-reported acne with lack of a physician verified diagnosis of acne [15,16,17,20,25] may lead to misclassification bias as validity of self-reported acne is at best only moderate, with sensitivity of 55%, specificity of 72%, positive predictive value of 70%, and negative predictive value of 57% [50]. Including other dermatology patients as controls [26] may attenuate associations, as seborrhea may play a role in several diseases. The observational studies were cross-sectional [20,21,46,47], case-control [18,22,23,24,26], retrospective [15], or longitudinal [16,17,25]; thus, in most studies we cannot rule out reverse causation. Questionnaire ascertainment of dairy intake varied between the articles and only a few studies used validated food frequency questionnaires [15,16,17,21]. Despite the food questionnaire used, participants may deliberately over- or underestimate (information bias) or not accurately remember (recall bias) when filling out questionnaires about dairy intake and acne. Furthermore, it was not possible to differentiate acne development, acne triggers, and severity of acne in the meta-analyses. Only a few studies provided adjusted results [15,16,17,25] so we based most of the analyses on raw numbers, which makes it difficult to rule out confounding from other dietary factors (e.g., glycemic index or calorie intake) or other lifestyle factors previously associated with acne [4,9,28]. 

Acne prevalence varied remarkably across the included studies, between 7–89%. The retrospective study by Adebamowo in 2005 with 7.3% acne cases focused on recall data provided by subjects in the Nurses’ Health Study II (NHS), which were aged 25–42 years old in 1989 when information on teenage acne was collected [15]; thus, the acne prevalence is likely underestimated and the results from this study may not be representative. Furthermore, the studies from 2006 and 2008 were offspring studies from the NHS in girls and boys [16,17]; however, leave-one-out analyses revealed that only the Adebamowo 2005 study was an outlier [15].

The observational studies may suffer from bias from confounding and reverse causation [9], are unable to indicate causality of the relationship between dairy and acne, and unable to prove preventive effects of abstaining from dairy. Only one study exists on milk intake and acne. The study is uncontrolled and unblinded and is based on medical students who drank milk or consumed other potential acne provoking foods. In addition, the total number of people with and without acne lesions were counted for all foods combined, but with no formal statistical testing [51]. Thus, there is still a knowledge gap with respect to whether dairy intake is causally associated with acne, acne flare, or acne severity and to what extent. To answer this question, we would ideally need results from large clinical randomized double-blind placebo-controlled trials (RCT); however, the question is whether this is realistically possible ethically, clinically, and/or operationally. Another approach (which no previous studies have yet undertaken) would be to perform a Mendelian Randomization study of lactase persistence, dairy intake, and acne using genetic lactase persistence as a proxy for lifetime dairy intake under the assumption that alleles are randomly distributed at conception [52,53]. Such a study design mimics an RCT and allows for the causal estimate of dairy intake and acne.

The observational studies all assessed dairy intake as an isolated factor. However, dairy is part of various individual and cultural specific diets and not a single factor with a single factor prediction (“reductionist approach” [54]). Instead, other factors which can affect the bioactive properties of nutrients in dairy and milk intake should be taken into consideration, such as macro- and micronutrients (fat, protein, carbohydrates, vitamins, sodium, and minerals), the dairy structure (liquid or solid), fermentation, and processing (holistic approach [55]). Only two studies in the meta-analysis also reported the glycemic load and glycemic indices of food consumed in conjunction with milk/dairy products [18,22], but did not report the glycemic load from the dairy consumption specifically. Hyperglycemic carbohydrates enhance insulin signaling, which promotes insulin and IGF-1 signaling, which in a synergistic fashion with milk stimulate mTORC1(mammalian target of rapamycin complex 1) signal transduction [56]. There is accumulating evidence that acne belongs to the spectrum of mTORC1-driven diseases of civilization including metabolic syndrome, obesity, insulin resistance, and cancer [57]. A randomized trial has shown that a low-glycemic-load diet improves symptoms in acne vulgaris patients [58]. Interestingly, no acne was observed in the Kitavan Islanders (Pacific Ocean) and in the Ache Hunter-Gatherers from Paraguay, who live under Paleolithic conditions without milk/dairy and hyperglycemic food, although it should be acknowledged that many other differences exist to Western societies [59]. To present the pathological effects of milk in the Western diet it is therefore important to provide controlled studies that consider milk consumption in association with glycemic load and index as part of a mixed diet [60]. 

Recently, a meta-analysis of dairy and acne was published [29] but with several methodological flaws, including the inadvertent double-counting of studies (Landro [19]/Grossi [23], and Tsoy [30]/Tsoy [31]) due to duplicate publications, which caused inappropriate weighting of results and skewed pooled estimates. Using the double-counted studies by Tsoy, the authors also only used the most severe category of acne, which caused extremely high odds ratios of 10 and 12 to be included in the meta-analysis, further skewing the pooled estimates. Furthermore, the meta-analysis included a study by Agamia [41], which we decided to exclude as the intake of “milk and dairy produce” was poorly defined as “low” and “high” intake but not defined with any frequency, type, or amount of milk. The previous meta-analysis also did not provide evidence for the exact search strategy to be replicated, for the bias assessment using the Newcastle Ottawa scale, for leave-one-out analyses, or funnel plots of publication bias. As a comparison, in our meta-analysis, we included the exact search string so it can be replicated, the heterogeneity across studies, a bias assessment using the Newcastle-Ottawa scale presented with a table, a stratified analysis by study characteristics presented in figures, the details of the “leave-one-out” analysis presented in figures, and the publication bias presented in figures. Furthermore, we excluded duplicate studies, and we included four more papers [24,26,27,47] that were not included in the previous meta-analysis but should have been as the studies were published before the search for the previous meta-analysis was done in August 2017 [29]. It is of crucial importance that authors of meta-analyses have a critical judgement of the reliability and validity of the papers they consider including in a meta-analysis, otherwise the conduct and assessment of systematic reviews may be hampered.

## 5. Conclusions

In conclusion, this meta-analysis of observational studies has provided new insight into the direction and magnitude of the association between dairy intake and acne overall and by dairy type, amount, and frequency. It has shed light on the knowledge gaps and the limitations of the studies included compared to previous systematic and narrative reviews with no meta-analysis, heterogeneity assessment, or bias assessment included [4,9,28].

## Figures and Tables

**Table 1:** Characteristics of included studies for the association of dairy intake with acne in children, adolescents, and young adults.

First Author	Year	Study Population	Design	Age, Year	Gender	Country	Total *n*	Acne *n*	Acne (%)	No Acne *n*	Milk Variables	Acne Diagnosis
Adebamowo [15]	2005	Population cohort (Nurses’ Health Study II)	Retrospective	13–18	F	USA	46,879	3412	7.28	43,467	Any milk, whole milk, low-fat milk, skim milk	Q
Adebamowo [17]	2006	Population cohort (GUTS)—offspring of women in the Nurses’ Health Study II	Follow-up	9–15	F	USA	3756	2588	68.9	1168	Any milk, whole milk, low-fat milk, skim milk	Q
Adebamowo [16]	2008	Population cohort (GUTS)—offspring of women in the Nurses’ Health Study II	Follow-up	9–15	M	USA	2759	1856	67.3	903	Any milk, whole milk, low-fat milk, skim milk	Q
Cerman [22]	2016	Acne patients and healthy controls	Case-control	19	F/M	Turkey	86	50	58.1	36	Any milk	D
Duquia [46]	2017	Acne patients vs. healthy controls in the army	Cross-sectional	18	M	Brasil	2201	1960	89.1	241	Whole milk, low-fat milk, cheese, yogurt	P
Grossi [23]	2016	Acne patients and non-acne dermatology patient controls	Case-control	10–24	F/M	Italy	563	205	36.4	358	Any milk, whole milk, skim milk, cheese/yogurt combined	D
Ismail [18]	2012	Acne patients and healthy controls	Case-control	18–30	F/M	Malaysia	88	44	50	44	Any milk, yogurt, cheese	D
Karadag [26]	2017	Acne patients and non-acne dermatology patient controls	Case-control	21	M	Turkey	4595	3836	83.5	759	Milk/cheese combined	D
Jung [24]	2010	Acne patients and age-matched healthy controls	Case-control	24	F/M	South Korea	1285	783	60.9	502	Cheese	D
Okoro [21]	2016	Population cohort	Cross-sectional	11–30	F/M	Nigeria	450	292	64.9	158	Any milk	D
Park [47]	2015	Population cohort	Cross-sectional	7–12	F/M	South Korea	693	251	36.2	442	Any milk, cheese, yogurt	D
Ulvestad [25]	2016	Population cohort	Follow-up	15–19	F/M	Norway	2387	331	13.9	2056	Any milk, full-fat milk	Q
Wolkenstein [20]	2015	Population cohort	Cross-sectional	15–24	F/M	France	2266	1375	60.7	891	Any milk	Q
Wolkenstein [27]	2017	Population cohort	Cross-sectional	15–24	F/M	Europe *	10521	6063	57.6	4458	Whole milk, semi-skimmed milk, low-fat milk, dairy	Q

Age: mean or range. Q: Questionnaire. D: Dermatologist verified. GUTS: Growing Up Today Study. P: Physician verified. USA: United States of America. * 7 countries: Belgium, Czech and Slovak Republics, France, Italy, Poland, and Spain.

## Supplementary Materials

The following are available online at http://www.mdpi.com/2072-6643/10/8/1049/s1, Figure S1: Meta-analysis of full-fat dairy intake vs. no dairy intake, Figure S2: Meta-analysis of any milk intake vs. no milk intake, Figure S3: Meta-analysis of whole milk intake vs. no milk intake, Figure S4: Meta-analysis of low-fat/skim milk intake vs. no milk intake, Figure S5: Meta-analysis of Cheese intake vs. no cheese intake, Figure S6: Meta-analysis of yogurt intake vs. no yogurt intake, Figure S7: Meta-analyses of frequency of milk intake and acne, Figure S8: Meta-analyses of amount of total milk intake, Figure S9: Meta-analyses of amount of whole milk intake, Figure S10: Meta-analyses of amount of low-fat/skim milk intake, Figure S11: Meta-analyses of any dairy intake vs. no dairy intake—by age group, Figure S12: Meta-analyses of any dairy intake vs. no dairy intake—by gender group, Figure S13: Meta-analyses of any dairy intake vs. no dairy intake—by number of cases, Figure S14: Meta-analyses of any dairy intake vs. no dairy intake—by continent, Figure S15: Meta-analyses of any dairy intake vs. no dairy intake—by design, Figure S16: Meta-analyses of any dairy intake vs. no dairy intake—by acne diagnosis, Figure S17: Meta-analyses of any dairy intake vs. no dairy intake—by reference group, Figure S18: Meta-analysis of any milk intake vs. no milk intake—adjusted analysis, Figure S19: Meta-analysis of whole milk intake vs. no milk intake—adjusted analysis, Figure S20: Meta-analysis of low-fat/skim milk intake vs. no milk intake—adjusted analysis, Figure S21: Meta-analysis of any dairy intake vs. no dairy intake—leave one out analysis, Figure S22: Meta-analysis of any milk intake vs. no milk intake—leave one out analysis, Figure S23: Funnel plot of standard error by log odds ratio—any dairy intake vs. no dairy intake, Figure S24: Funnel plot of standard error by log odds ratio—any milk intake vs. no milk intake, Table S1: Sensitivity analyses for the association of dairy intake and acne, Table S2: Study-specific Newcastle-Ottawa quality assessment.
