# Supplementary material for: Dairy Intake and Acne Vulgaris: A Systematic Review and Meta-Analysis of 78,529 Children, Adolescents, and Young Adults
Source: Nutrients. 2018 Aug 9;10(8):1049. doi: 10.3390/nu10081049 (PMC6115795; doi:10.3390/nu10081049)
Supplement: Supplementary file 1 [file nutrients-10-01049-s001.pdf]

Supplementary Figure 1 Full-fat dairy intake vs. no dairy intake

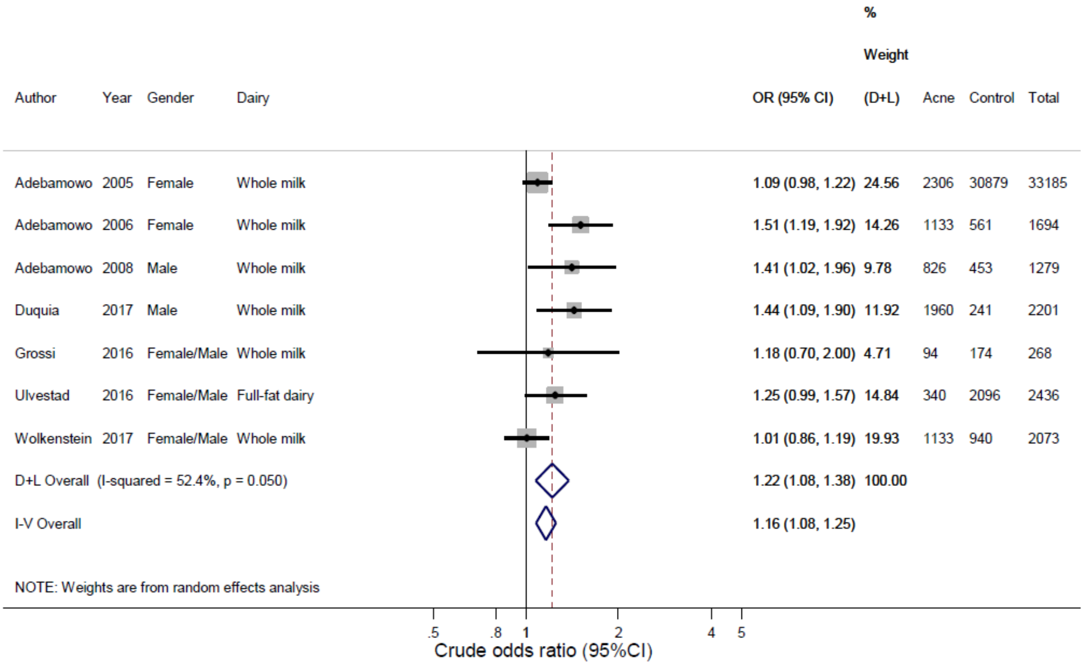

Supplementary Figure 2

Any milk intake vs. no milk intake

Author	Year	Gender	Dairy	OR (95% CI)	% Weight (D+L)	Acne	Control	Total
Adebamowo	2005	Female	Total milk	1.15 (1.03, 1.28)	18.75	3412	43467	46879
Adebamowo	2006	Female	Total milk	1.59 (1.27, 1.98)	12.80	2588	1168	3756
Adebamowo	2008	Male	Total milk	1.53 (1.13, 2.09)	9.07	1856	903	2759
Cerman	2016	Female/Male	Total milk	0.95 (0.40, 2.25)	1.85	50	36	86
Duquia	2017	Male	Total milk	1.36 (1.06, 1.75)	11.43	1960	241	2201
Grossi	2016	Female/Male	Total milk	1.22 (0.77, 1.94)	5.30	204	356	560
Ismail	2012	Female/Male	Total milk	3.99 (1.39, 11.43)	1.27	44	44	88
Okoro	2016	Female/Male	Total milk	1.62 (1.03, 2.56)	5.45	292	158	450
Park	2015	Female/Male	Total milk	0.96 (0.69, 1.36)	8.11	251	442	693
Wolkenstein	2015	Female/Male	Total milk	1.36 (0.91, 2.03)	6.45	1375	891	2266
Wolkenstein	2017	Female/Male	Total milk	1.08 (0.99, 1.19)	19.52	6063	4458	10521
D+L Overall (I-squared = 57.6%, p = 0.009)				1.28 (1.13, 1.44)	100.00			
I-V Overall				1.18 (1.11, 1.25)				

NOTE: Weights are from random effects analysis

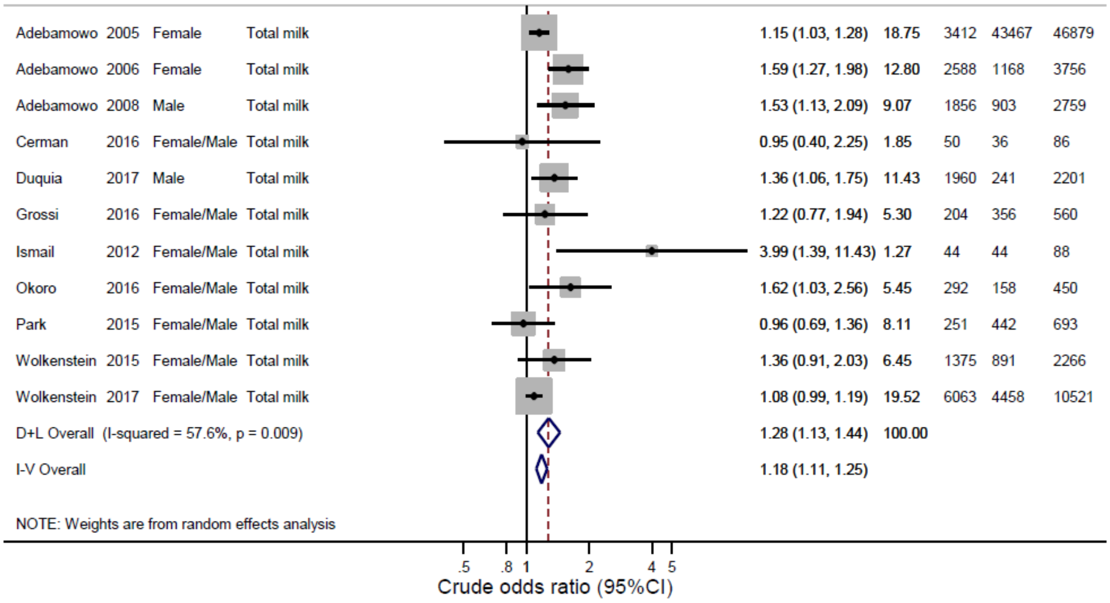

Supplementary Figure 3

Whole milk intake vs. no milk intake

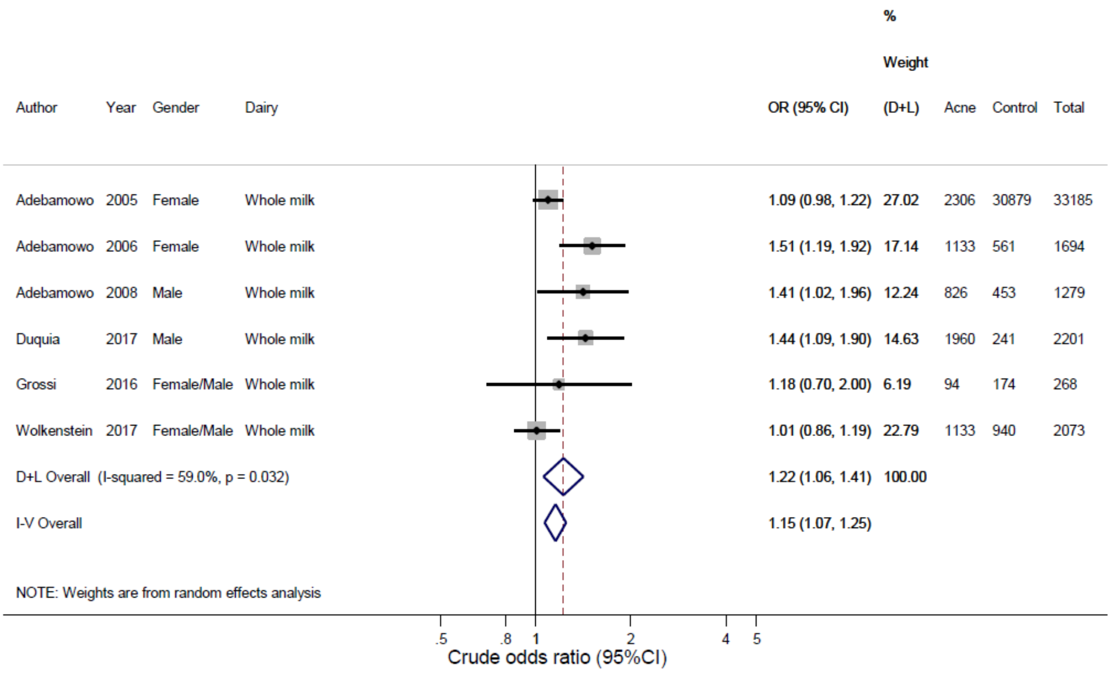

Supplementary Figure 4

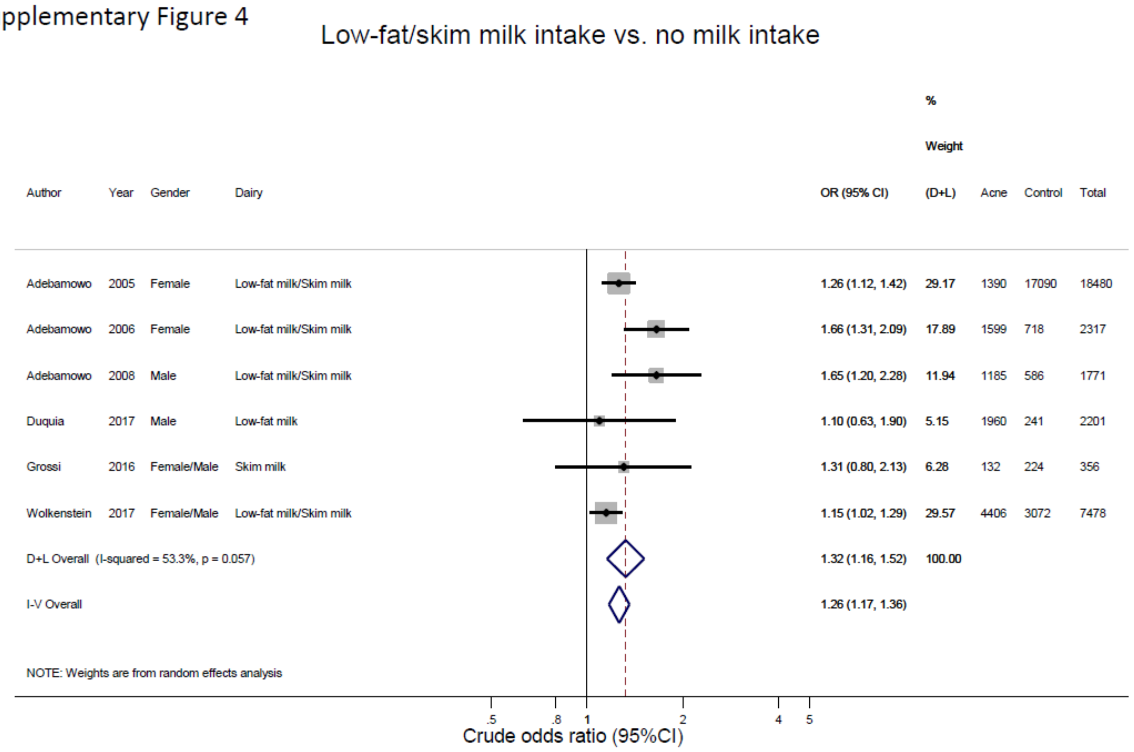

Supplementary Figure 5

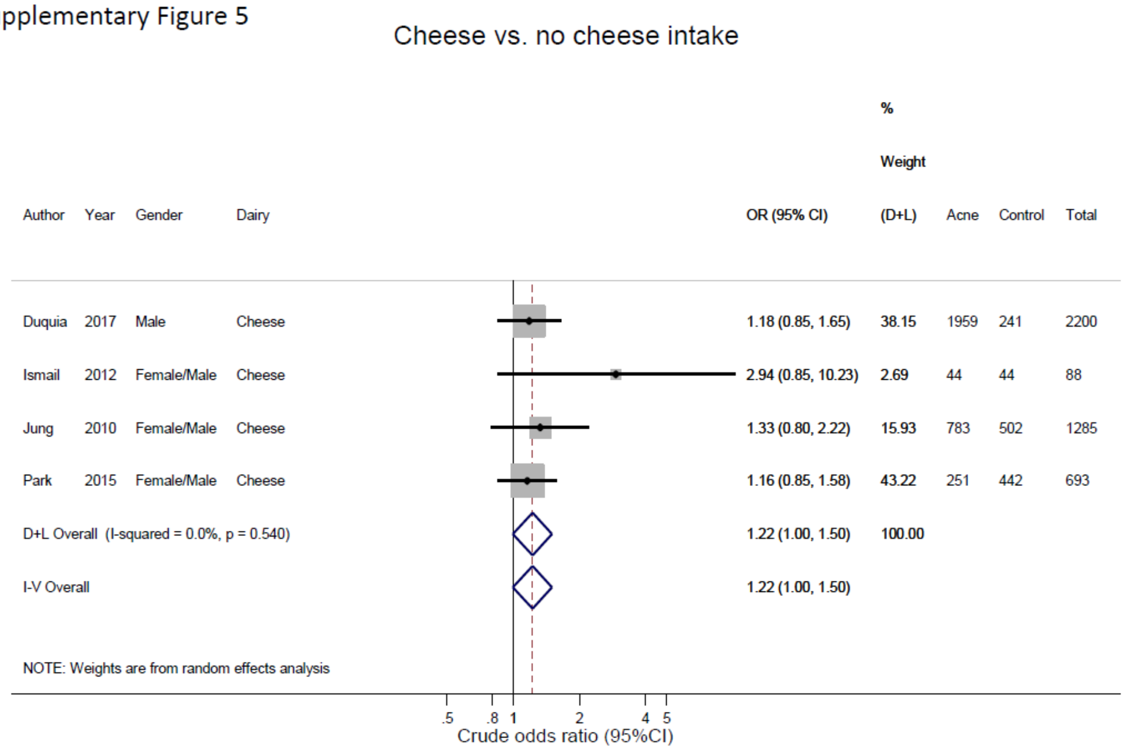

Supplementary Figure 6

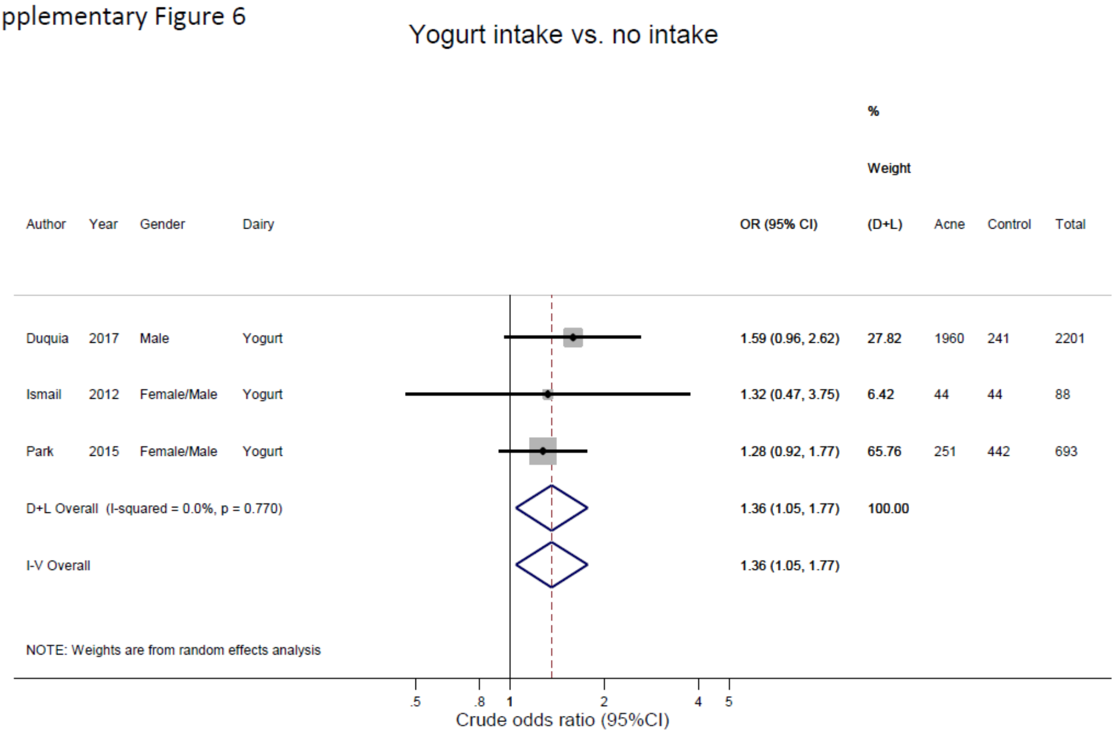

Supplementary Figure 7

Meta-analyses of frequency of milk intake and acne

compared to <=1 glass per week

Dose	Studies(N)	Acne(N)	Controls(N)	Total(N)	pvalue(het)	I2(%)	OR (95% CI)	pvalue
Any milk								
2-6 glasses per week	3	2197	17919	20116	0.02	75	1.24 (0.95, 1.62)	1.10e-01
1 glass per day	3	2237	14640	16877	0.01	79	1.41 (1.05, 1.90)	2.41e-02
≥ 2 glasses per day	3	4750	26859	31609	0.01	80	1.43 (1.09, 1.88)	9.69e-03
.								
Whole milk								
2-6 glasses per week	3	1649	13971	15620	0.02	76	1.23 (0.90, 1.69)	1.95e-01
1 glass per day	3	1518	11665	13183	0.08	60	1.29 (1.01, 1.66)	4.37e-02
≥ 2 glasses per day	3	2544	18441	20985	0.05	66	1.33 (1.05, 1.68)	1.75e-02
.								
Low-fat/skim milk								
2-6 glasses per week	3	1431	9490	10921	0.48	0	1.27 (1.11, 1.45)	4.32e-04
1 glass per day	3	1376	8751	10127	0.02	74	1.48 (1.07, 2.05)	1.89e-02
≥ 2 glasses per day	3	2712	12261	14973	0.06	66	1.54 (1.23, 1.93)	1.85e-04
.								

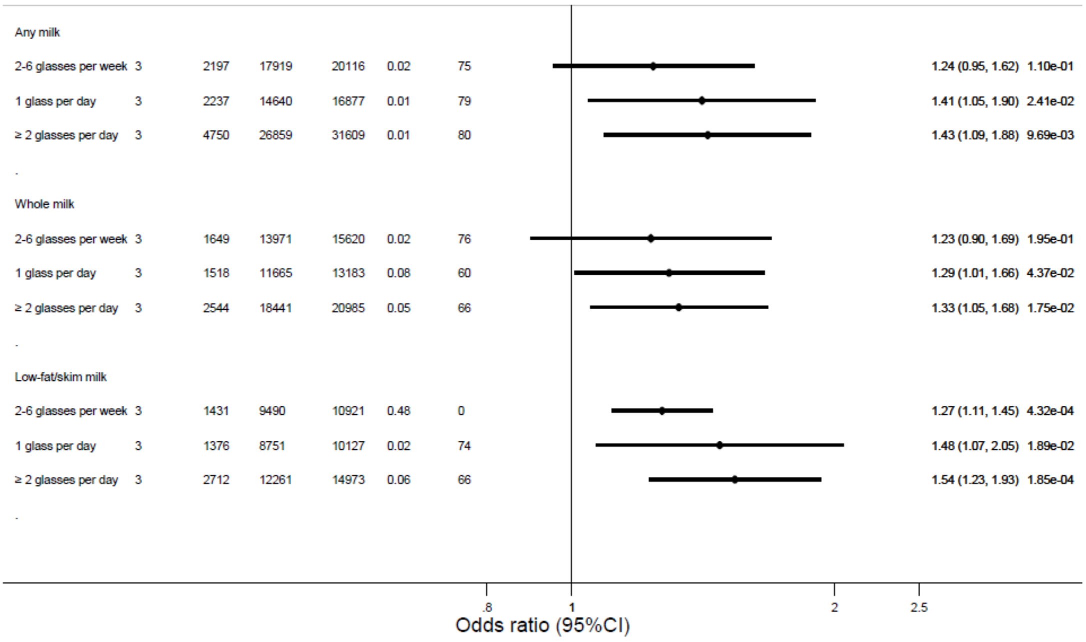

Supplementary Figure 8

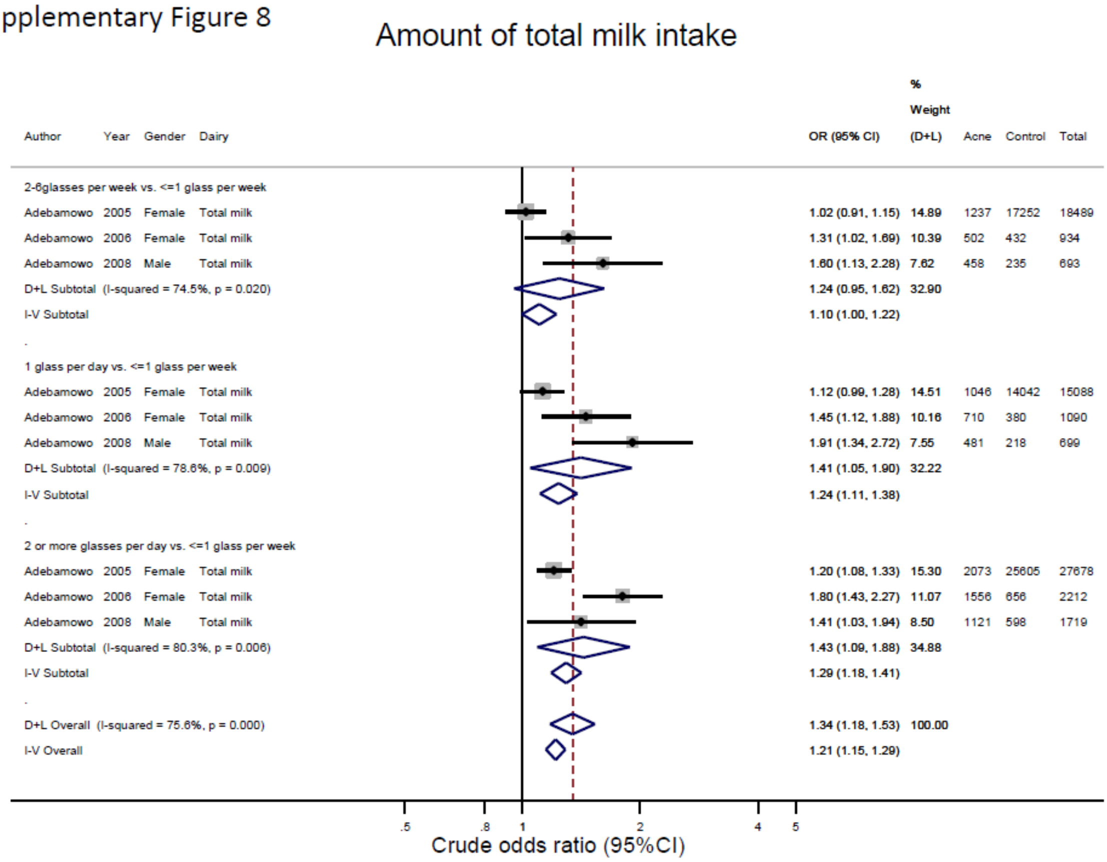

Supplementary Figure 9

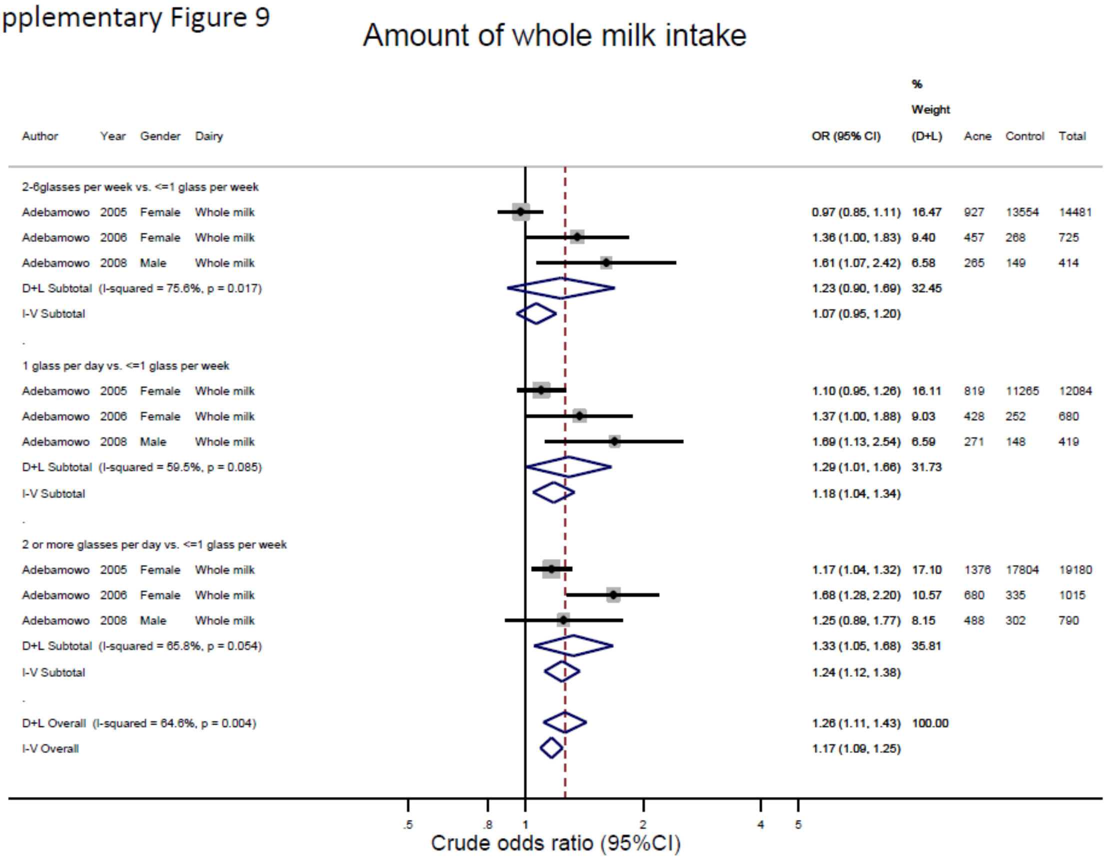

Supplementary Figure 10

Amount of low-fat/skim milk intake

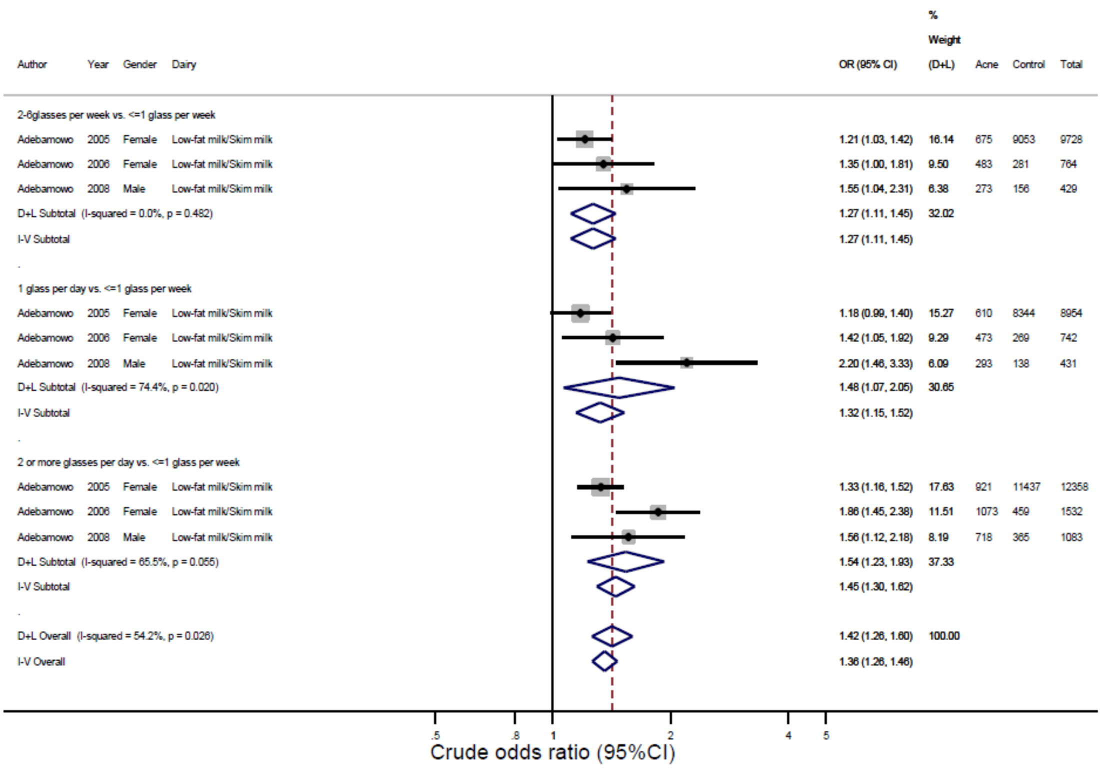

Supplementary Figure 11

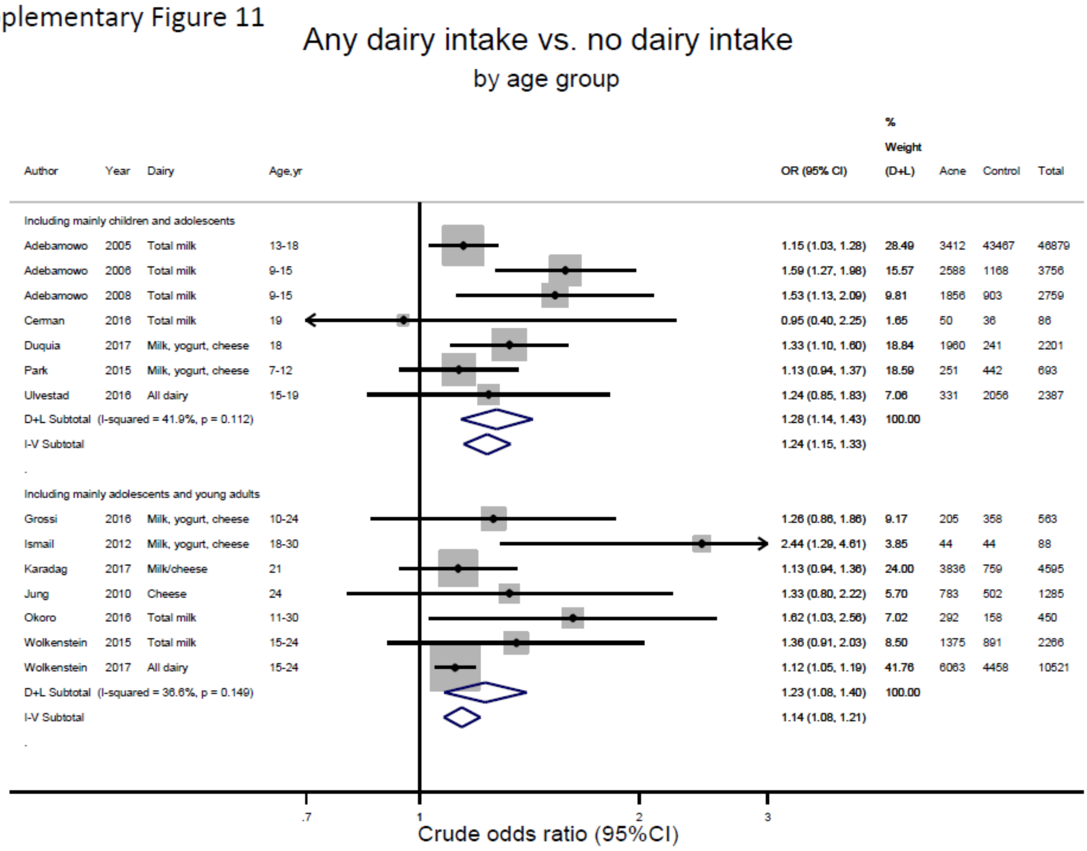

Supplementary Figure 12

Any dairy intake vs. no dairy intake

by gender group

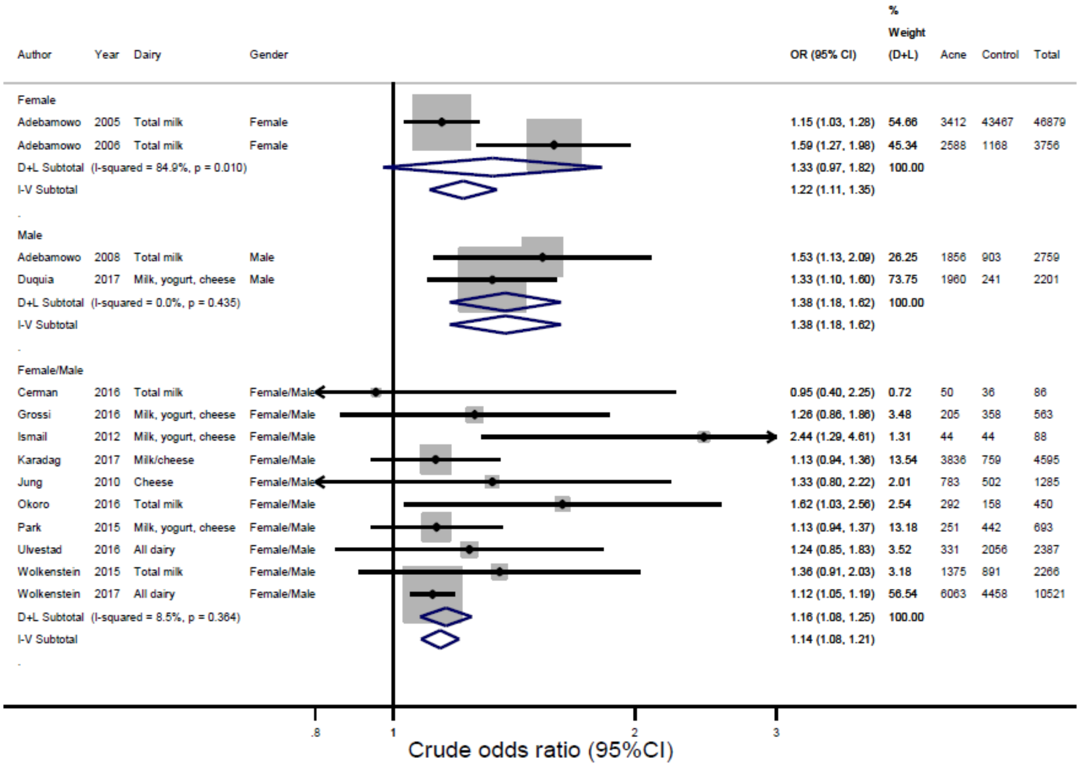

Supplementary Figure 13

Any dairy intake vs. no dairy intake

by number of cases

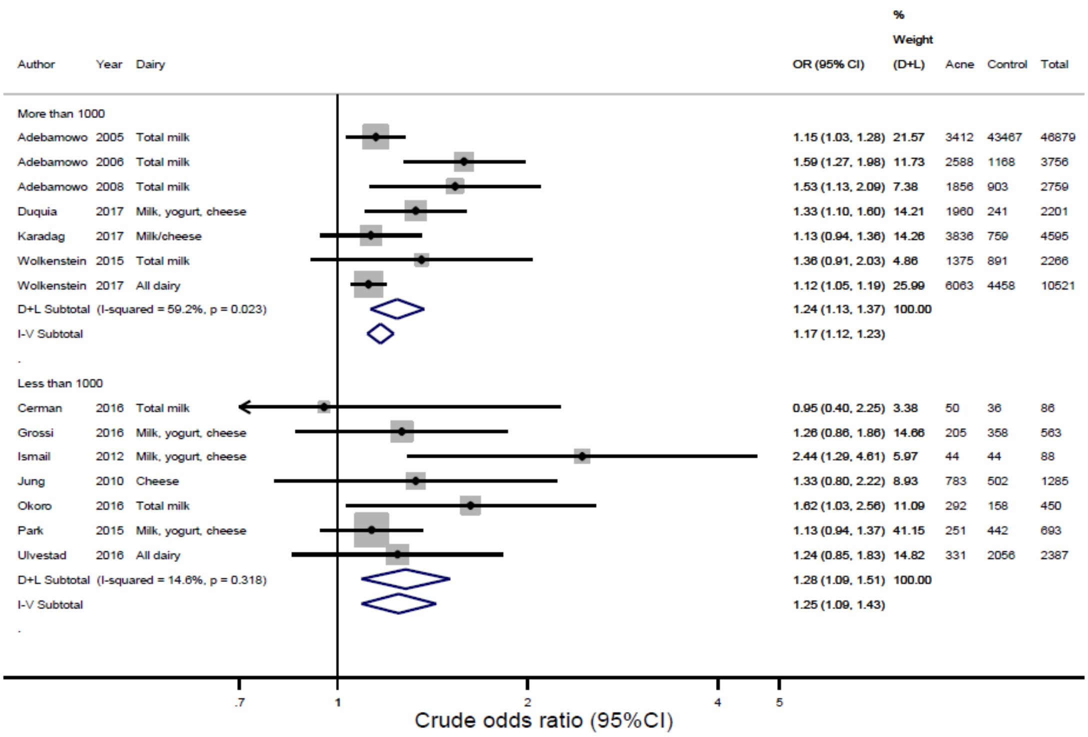

Supplementary Figure 14

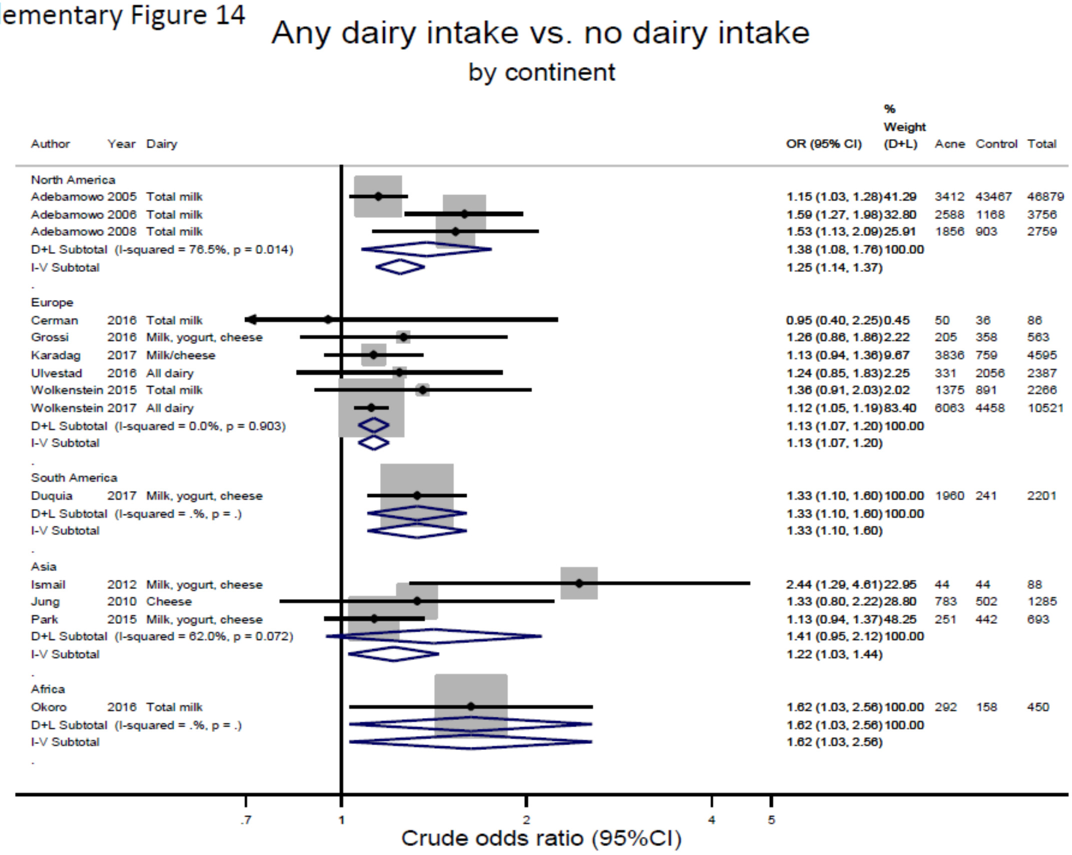

Supplementary Figure 15

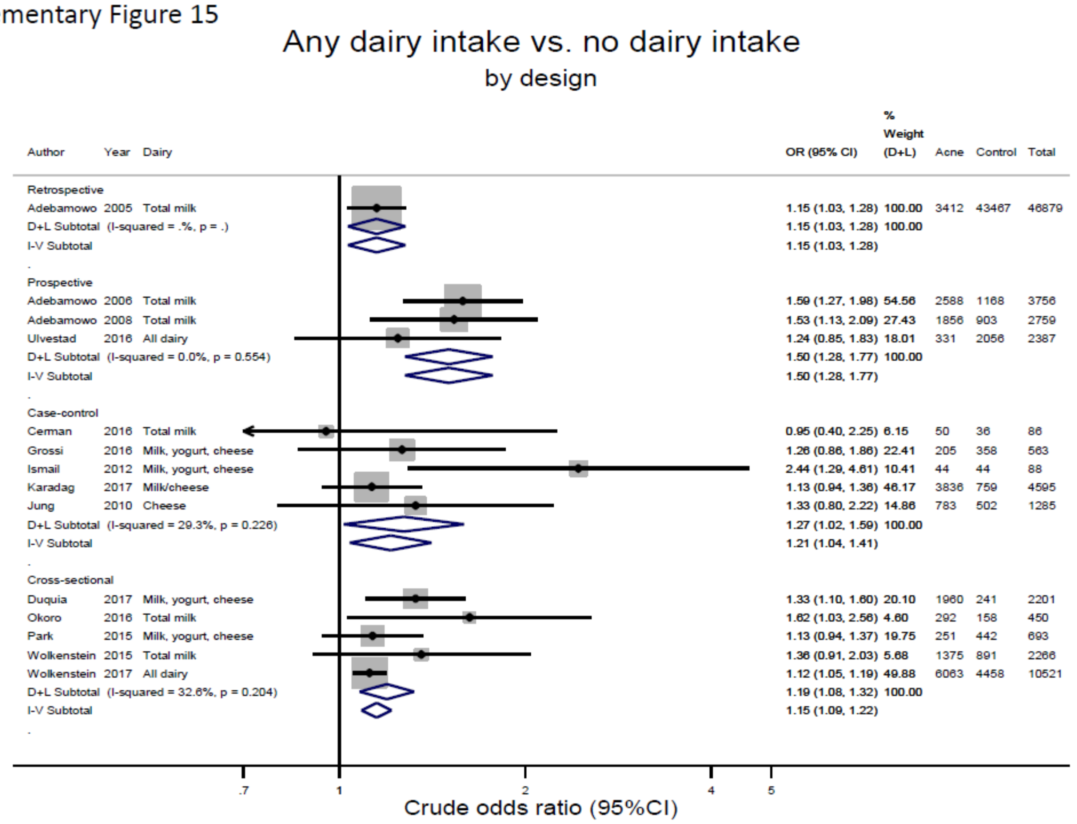

Supplementary Figure 16

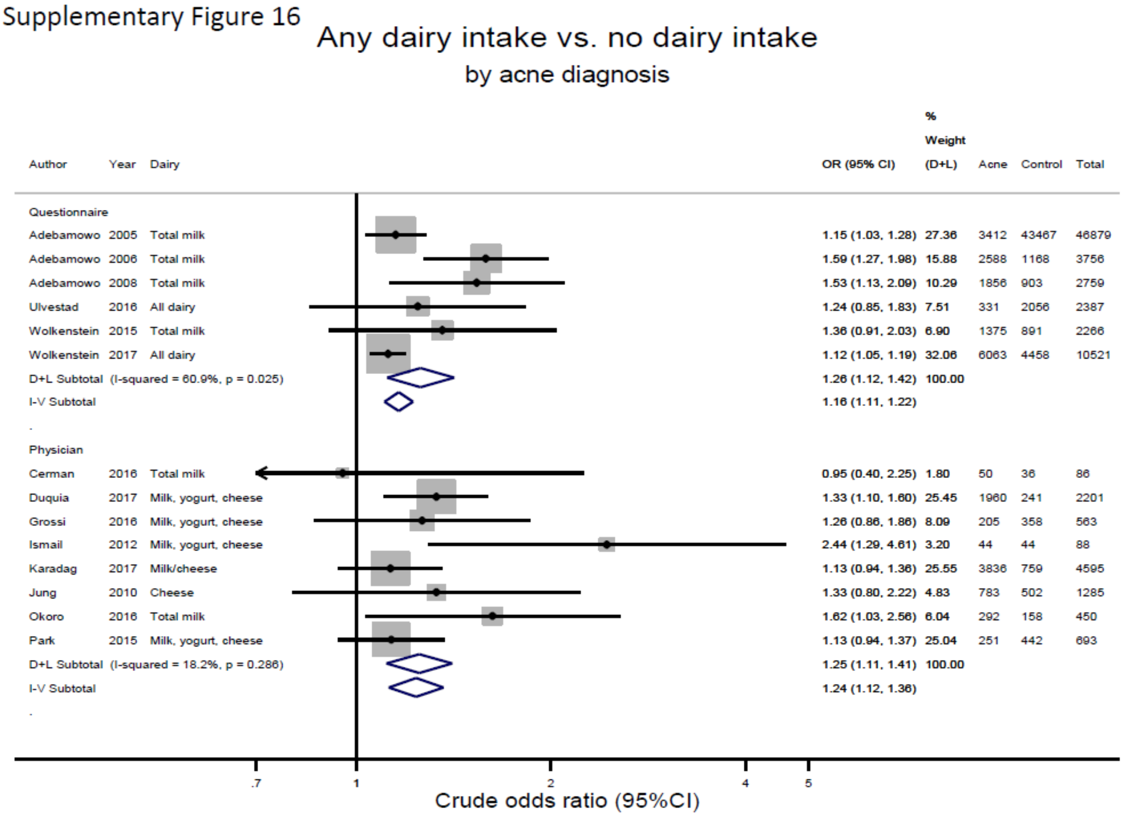

Supplementary Figure 17

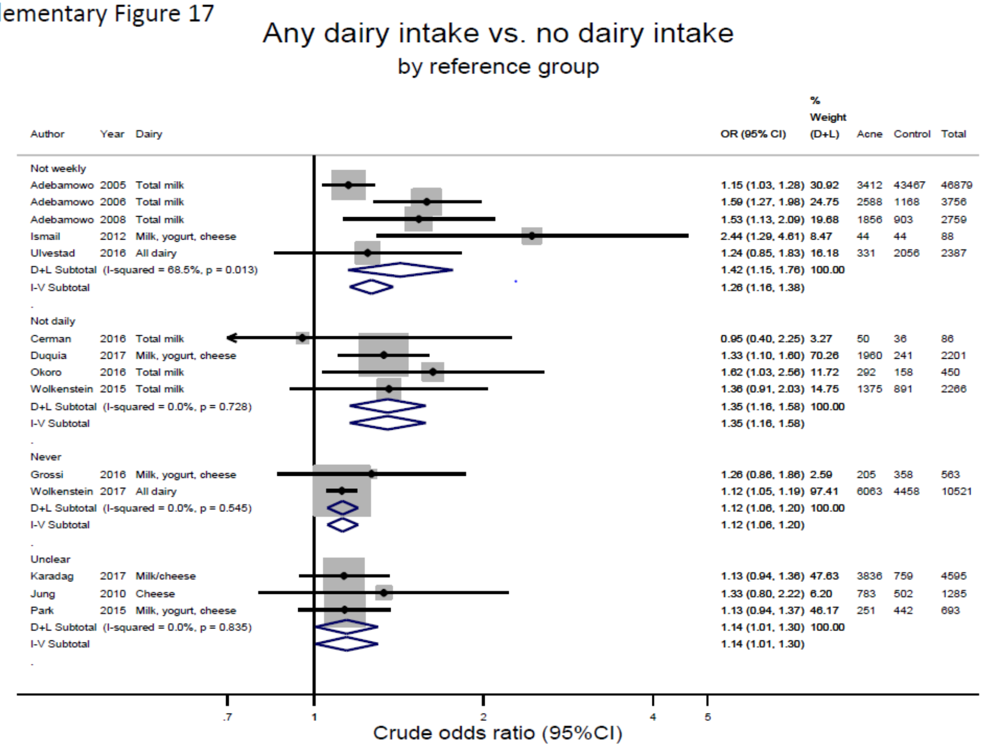

Supplementary Figure 18

Any milk intake vs. no milk intake

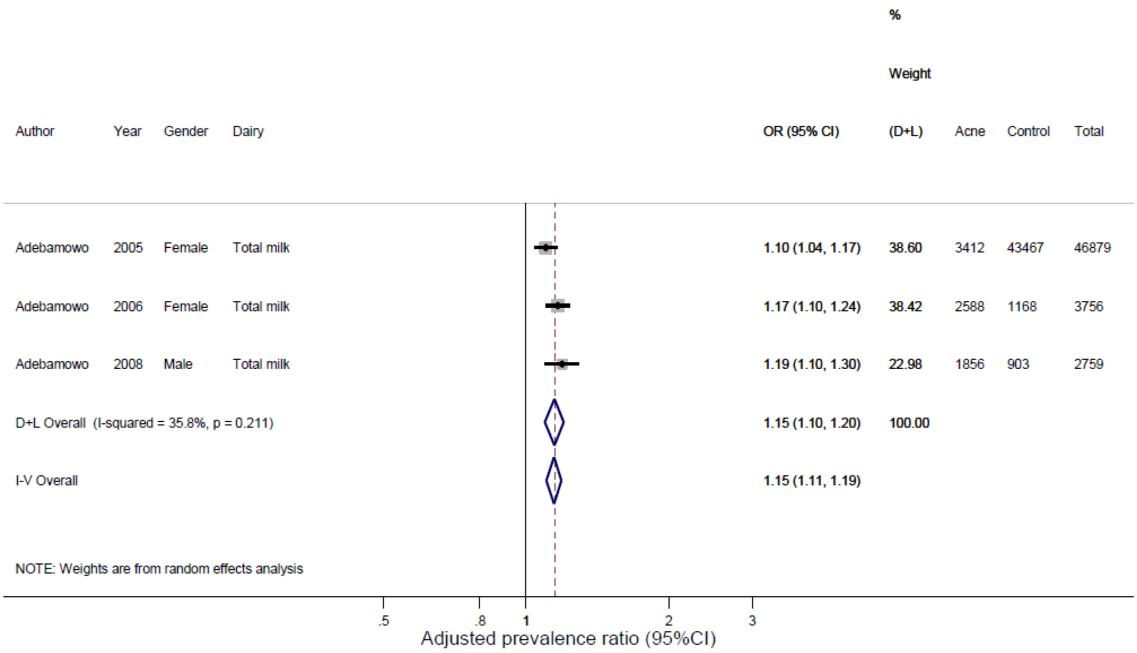

Supplementary Figure 19

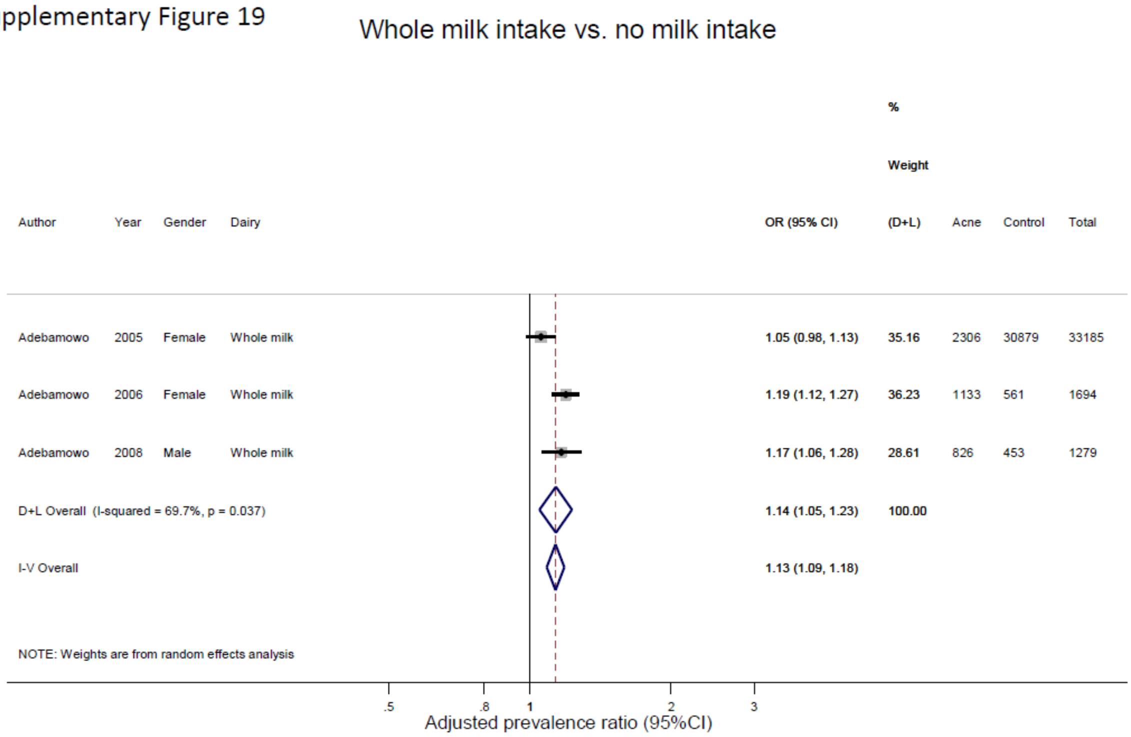

Supplementary Figure 20

Low-fat/skim milk intake vs. no milk intake

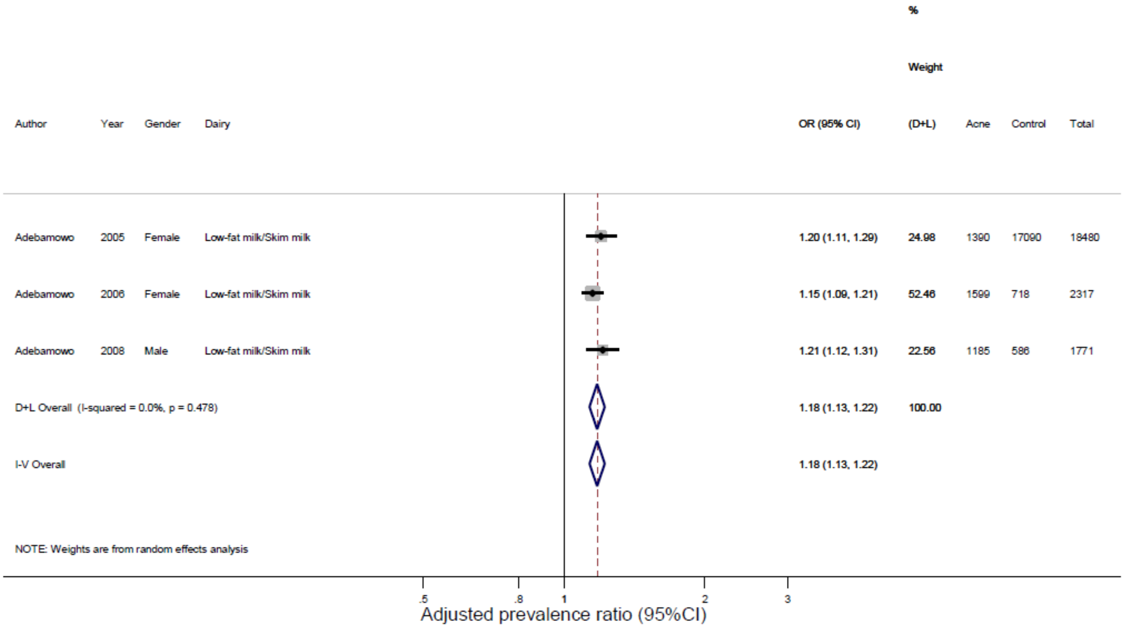

Supplementary Figure 21

Leave one out analysis
- Any dairy intake vs. no dairy intake

Study name	Statistics with study removed				
	Point	Lower limit	Upper limit	Z-Value	p-Value
Adebamowo, 2005	1.284	1.164	1.417	4.992	0.000
Adebamowo, 2006	1.198	1.121	1.282	5.275	0.000
Adebamowo, 2008	1.232	1.138	1.334	5.136	0.000
Cerman	1.256	1.156	1.365	5.385	0.000
Duquia	1.243	1.140	1.355	4.936	0.000
Grossi	1.253	1.151	1.364	5.215	0.000
Ismail	1.225	1.140	1.315	5.542	0.000
Karadag	1.272	1.163	1.392	5.246	0.000
Jung	1.251	1.151	1.361	5.245	0.000
Okoro	1.239	1.143	1.342	5.228	0.000
Park	1.271	1.162	1.391	5.240	0.000
Ulvestad	1.254	1.152	1.366	5.224	0.000
Wolkenstein, 2015	1.249	1.148	1.358	5.192	0.000
Wolkenstein, 2017	1.285	1.175	1.406	5.466	0.000
	1.250	1.153	1.356	5.416	0.000

Odds ratio (95% CI) with study removed

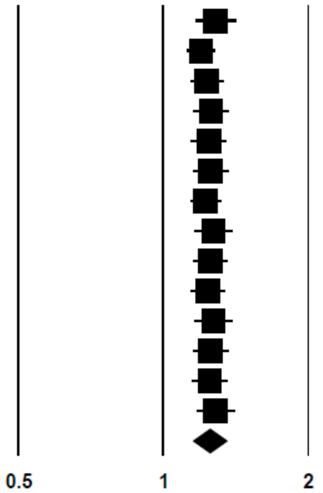

Supplementary Figure 22

Leave one out analysis
- Any milk intake vs. no milk intake

Study name	Statistics with study removed				
	Point	Lower limit	Upper limit	Z-Value	p-Value
Adebamowo, 2005	1.324	1.128	1.555	3.429	0.001
Adebamowo, 2006	1.222	1.091	1.369	3.455	0.001
Adebamowo, 2008	1.253	1.105	1.420	3.529	0.000
Cerman	1.288	1.135	1.460	3.940	0.000
Duquia	1.271	1.112	1.452	3.528	0.000
Grossi	1.285	1.129	1.462	3.791	0.000
Ismail	1.252	1.120	1.399	3.963	0.000
Okoro	1.260	1.113	1.426	3.643	0.000
Park	1.312	1.153	1.491	4.136	0.000
Wolkenstein, 2015	1.275	1.120	1.451	3.683	0.000
Wolkenstein, 2017	1.333	1.157	1.537	3.966	0.000
	1.278	1.131	1.444	3.937	0.000

Odds ratio (95% CI) with study removed

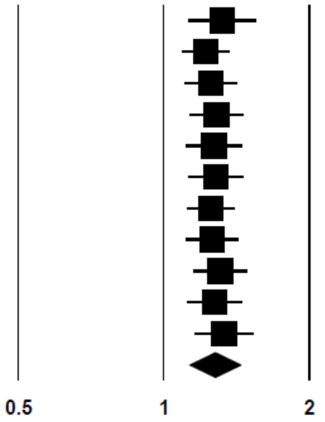

Supplementary Figure 23

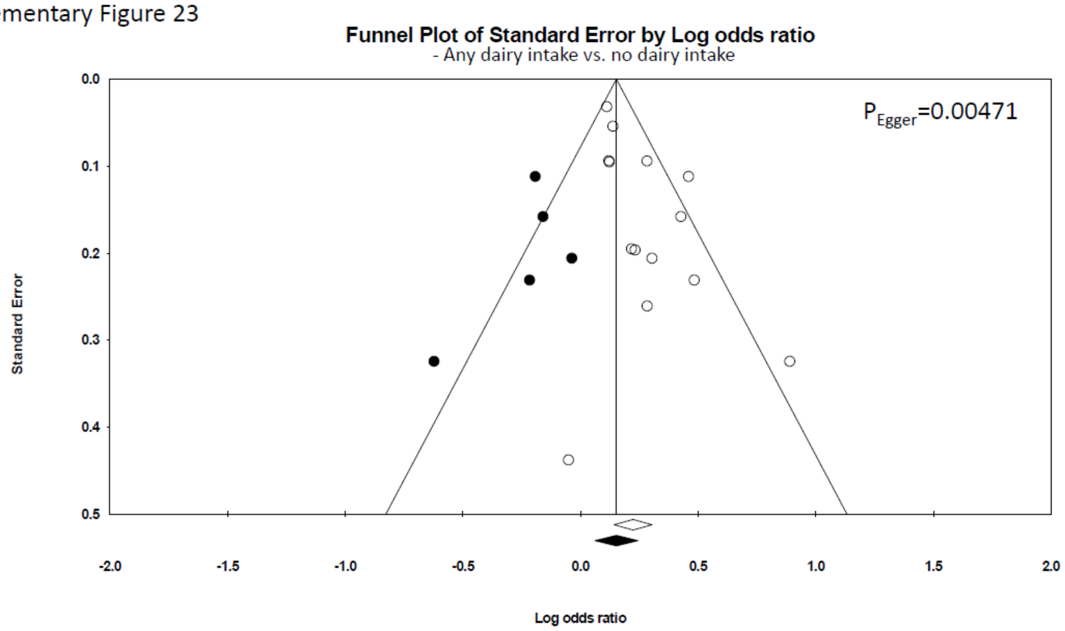

Supplementary Figure 24

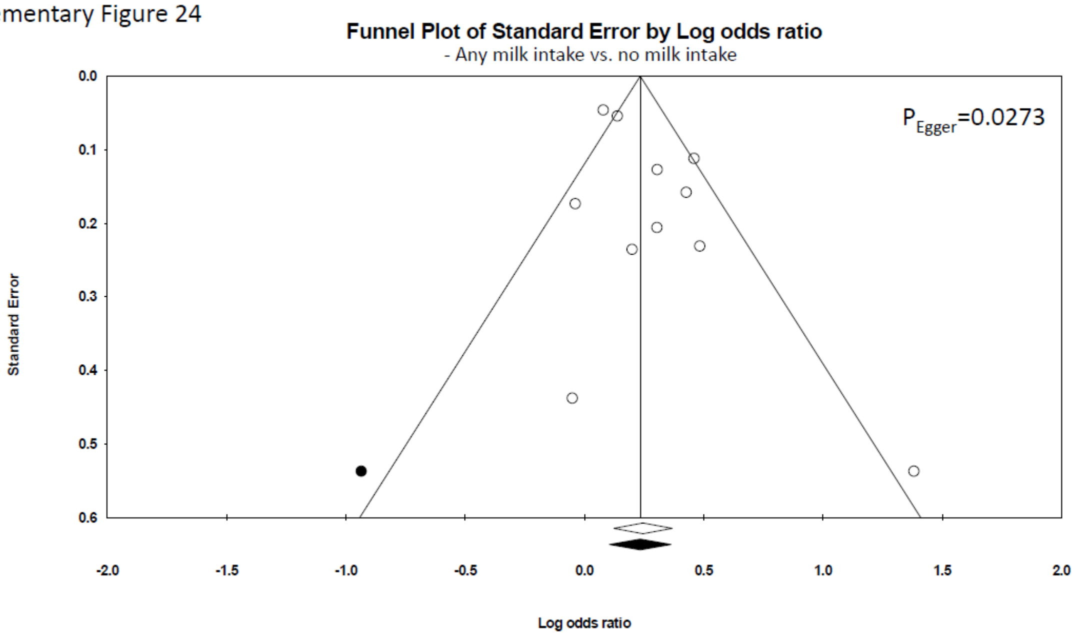

Supplementary Table 1. Sensitivity analyses for the assocation of dairy intake and acne

Subgroup	Studies,N	OR(95% CI)*	I-square,%	p(het)
Age				
Children and adolescents	7	1.28(1.14-1.43)	42	0.112
Adolescents and young adults	7	1.23(1.08-1.40)	37	0.149
Gender				
Female	2	1.33(0.97-1.82)	85	0.010
Male	2	1.38(1.18-1.62)	0	0.435
Female/male	10	1.16(1.08-1.25)	9	0.364
Cases				
More than 1000	7	1.24(1.13-1.37)	59	0.023
Less than 1000	7	1.28(1.09-1.51)	15	0.318
Continent				
North America	3	1.38(1.08-1.76)	77	0.014
Europe	6	1.13(1.07-1.20)	0	0.903
South America	1	1.33(1.10-1.60)	NA	NA
Asia	3	1.41(0.95-2.12)	62	0.072
Africa	1	1.62(1.03-2.56)	NA	NA
Design				
Retrospective	1	1.15(1.03-1.28)	NA	NA
Prospective	3	1.50(1.28-1.77)	0	0.554
Case-control	5	1.27(1.02-1.59)	29	0.226
Cross-sectional	5	1.19(1.08-1.32)	33	0.204
Acne Diagnosis				
Questioannaire	7	1.23(1.11-1.36)	53	0.045
Physician-verified	7	1.30(1.13-1.49)	19	0.286
Reference group				
Not weekly	5	1.42(1.15-1.76)	69	0.013
Not daily	4	1.35(1.16-1.58)	0	0.728
Never	2	1.12(1.06-1.20)	0	0.545
Unclear	3	1.14(1.01-1.30)	0	0.835

*Random effects meta-analyses

NA: not applicable

p(het): p-value for heterogeneity based on Q-statistics

Supplementary Table 2. Study-specific Newcastle-Ottawa quality assessment

Case-control studies		Selection				Comparability	Exposure			Total
		S1	S2	S3	S4	C1	E1	E2	E3	
		Case validation	Case representativeness	Control selection	Control validation	Confounder adjustment	Ascertainment	Method case/control	Non-response	
Cerman	2016	1	0	1	0	0	0	0	0	2
Grossi	2016	1	1	0	1	0	0	1	0	4
Ismail	2012	1	1	1	1	0	0	1	0	5
Karadag	2017	1	1	0	1	0	0	1	0	4
Jung	2010	1	1	1	1	0	0	1	0	5

Cohort studies		Selection				Comparability	Outcome			
		S1	S2	S3	S4	C1	O1	O2	O3	
		Exposed representativeness	Non-exposed Selection	Exposure ascertainment	Outcome baseline		Outcome assessment	Follow up	Losses follow-up	
Adebamowo	2004	1	1	0	0	2	0	0	0	4
Adebamowo	2006	1	1	0	0	2	0	1	1	6
Adebamowo	2008	1	1	0	0	2	0	1	1	6
Duquia	2017	1	1	0	0	1	0	0	0	3
Okoro	2016	1	1	0	0	0	0	0	0	2
Park	2015	1	1	0	0	0	0	0	0	2
Ulvestad	2016	1	1	0	0	2	0	1	1	6
Wolkenstein	2015	1	1	0	0	0	0	0	0	2
Wolkenstein	2017	1	1	0	0	0	0	0	0	2

Legend: A minimum number of one star (i.e. asterisk) is awarded within category "Selection", and a maximum of two stars within "Comparability". Answers "a" and/or "b" within the sub-categories S1-S4, C1, E1-E3, and O1-O3 meet the requirement for a star. Sub-categories evaluate definitions and selection of cases and controls, representation and selection of cohorts, ascertainment of exposure, and outcome.
